# Roles of Salivary Components in *Streptococcus mutans* Colonization in a New Animal Model Using NOD/SCID.*e2f1* ^−/−^ Mice

**DOI:** 10.1371/journal.pone.0032063

**Published:** 2012-02-21

**Authors:** Tatsuro Ito, Takahide Maeda, Hidenobu Senpuku

**Affiliations:** 1 Department of Pediatric Dentistry, Nihon University Graduate School of Dentistry at Matsudo, Chiba, Japan; 2 Department of Bacteriology, National Institute of Infectious Diseases, Tokyo, Japan; Centers for Disease Control & Prevention, United States of America

## Abstract

*Streptococcus mutans* plays an important role in biofilm formation on the tooth surface and is the primary causative agent of dental caries. The binding of *S. mutans* to the salivary pellicle is of considerable etiologic significance and is important in biofilm development. Recently, we produced NOD/SCID.*e2f1* ^−/−^ mice that show hyposalivation, lower salivary antibody, and an extended life span compared to the parent strain: NOD.*e2f1* ^−/−^. In this study we used NOD/SCID.*e2f1* ^−/−^ 4 or 6 mice to determine the roles of several salivary components in *S. mutans* colonization *in vivo*. *S. mutans* colonization in NOD/SCID.*e2f1^−/−^* mice was significantly increased when mice were pre-treated with human saliva or commercial salivary components. Interestingly, pre-treatment with secretory IgA (sIgA) at physiological concentrations promoted significant colonization of *S. mutans* compared with sIgA at higher concentrations, or with human saliva or other components. Our data suggest the principal effects of specific sIgA on *S. mutans* occur during *S. mutans* colonization, where the appropriate concentration of specific sIgA may serve as an anti-microbial agent, agglutinin, or an adherence receptor to surface antigens. Further, specific sIgA supported biofilm formation when the mice were supplied 1% sucrose water and a non-sucrose diet. The data suggests that there are multiple effects exerted by sIgA in *S. mutans* colonization, with synergistic effects evident under the condition of sIgA and limited nutrients on colonization in NOD/SCID.*e2f1* ^−/−^ mice. This is a new animal model that can be used to assess prevention methods for dental biofilm-dependent diseases such as dental caries.

## Introduction

Oral streptococci are present in large numbers in dental plaque, which co-interact with the enamel salivary pellicle to form a biofilm on tooth surfaces [1], [2]. Streptococcal cell wall components mediate adherence to various salivary receptors [3]–[6]. The ability of oral streptococci to bind to the salivary pellicle is of considerable etiologic significance in oral disease [4], [7]; and is important for biofilm development [8], [9]. The glucans synthesized by streptococcal glucosyltransferases convert sucrose into glucan; and provide binding sites through interaction with bacterial cell-associated glucan-binding proteins that promote the accumulation of microorganisms on the tooth surface, and help establish pathogenic biofilms [10], [11]. *Streptococcus mutans* plays an important role in biofilm formation on the tooth surface and is a primary causative agent for dental caries [2]. *S. mutans* produces two extracellular glucosyltransferase (Gtfs) that convert sucrose into insoluble glucans [10], where GTF I and GTF SI (water-insoluble glucan) are encoded by *gtfB* and *gtfC*. Animal experiments [12] suggest that the expression of these two *S. mutans gtf* genes is required for maximal virulence in causing dental caries.

It is difficult to extrapolate *in vitro* experimental results to predict the impact of a specific salivary factor in biofilm development. However, the problem facing *in vivo* oral biofilm research is the lack of a natural, reproducible, longitudinal monitoring system permitting the assessment of oral bacterial infection in the same animal throughout the duration of a study. Studies using *S. mutans* infection in animal oral cavities have been performed by feeding the animals powdered Diet 2000 containing unnatural amounts of sucrose (56%). Even when experiments employed feeding a low sucrose content (1 or 5%), longitudinal (more than 2 weeks) feeding with frequent inoculation was performed [13]–[17]. When these methods were used, *S. mutans* was found to produce a larger amount of insoluble glucan in the oral cavities of mice fed foods containing excess amounts of sucrose. These experiments although interesting do not represent human diet styles.

The mechanical forces of salivary flow and tongue movement tend to dislodge and expel bacteria from tooth surfaces and the oral cavity [18], [19]. This controls microbial colonization in the oral cavity as shown with insulin-dependent diabetes mellitus (IDDM), Sjögren's syndrome (SS), and drymouth where these patients suffer from a rapid overgrowth of biofilm and caries that make them highly susceptible to oral infections [20], [21]. E2F-1 is a member of the transcriptional factor controlling the initiation of DNA synthesis [22]–[24] and subsequent transition of cells from the G0/G1 to S phase in the cell cycle [25], [26]. Several recent studies have demonstrated that a mutation of the *e2f1* gene in mice causes enhanced T-lymphocyte proliferation, leading to testicular atrophy, splenomegaly, salivary gland dysplasia, and other types of systemic and organ-specific autoimmunity [27]–[30]. C57BL/6.*e2f1* ^−/−^ mice show high susceptibility to oral streptococci because they do not produce sufficient volumes of saliva and salivary proteins [31]. Further, the combination of E2F-1 deficiency and the NOD gene background induced a rapid progressive development of IDDM and SS compared to NOD mice. This is caused by enhanced auto-reactive Th1-type T cells. NOD.*e2f1* ^−/−^ mice do not survive long; therefore they are not suitable for long-term bacterial infection experiments [32]. A recent study using NOD/SCID background E2F-1 deficient mice (NOD/SCID.*e2f1* ^−/−^) (T and B cells do not develop to observe E2F-1 function in the NOD background mice without an auto-reactive response) showed E2F-1 may be associated with the differentiation of exocrine cells in the salivary duct [33].

The NOD/SCID.*e2f1* ^−/−^ mouse has a decreased saliva volume, lacks sIgA and IgG in the saliva, and has decreased NK cells. This may be a useful mouse for studying oral bacterial infection, colonization, and biofilm formation. These mice have long survival because they do not develop IDDM and SS. Therefore, they may be useful as a model animal for oral bacterial colonization under humanized conditions. Establishment of a humanized experimental system could lead to better understanding of the pathogenic conditions associated with oral bacterial infections and the development of more effective agents for control of bacterial infection associated with oral diseases.

## Materials and Methods

### Bacterial strains and culture conditions

*Streptococcus mutans* UA159 was used for colonization study and ELISA. *Actinomyces naeslundii* X600 was used for ELISA as control oral bacteria. All bacteria were grown in an atmosphere of H_2_ and CO_2_ (GasPack, Becton/Dickinson, Sparks, MD) in Brain Heart Infusion broth (BHI, Difco Laboratory, Detroit, MI) at 37°C.

### Animals

NOD/LtJ mice naturally develop IDDM, SS, and dry mouth; and were the parent strain to develop NOD/SCID.*e2f1* ^−/−^ mice. They were used as the control to compare *S. mutans* susceptibility to NOD back ground E2F-1^−/−^ mice (NOD.*e2f1* ^−/−^) and NOD/SCID back ground E2F-1 heterogeneous (NOD/SCID.*e2f1* ^+/−^) and homogeneous deficient NOD/SCID mice (NOD/SCID.*e2f1* ^−/−^) [33]. NOD/SCID mice were the parental lines to produce NOD/SCID.*e2f1* ^−/−^ mice [33] and were used as control mice in bacterial inoculation experiments. Heterozygous NOD/SCID.*e2f1* ^+/−^ mice were bred to generate NOD/SCID.*e2f1* ^−/−^ mice. Three types (+/+, +/− and −/− of *e2f1*) of NOD/SCID mice were screened using PCR [33]. All strains were female, 4 months of age and were maintained in accordance with the guidelines for the Care and Use of Laboratory Animals from the National Institute of Infectious Diseases. Experimental protocols (#209125, 210110, and 21124) were approved by the National Institute of Infectious Diseases Animal Resource Committee.

### Human saliva collection

Saliva samples were collected from volunteers with good oral health, after stimulation by chewing paraffin gum. The volunteers refrained from eating, drinking, and brushing for at least 2 h prior to collection. The saliva was placed into ice-chilled sterile bottles for 5 min; then centrifuged at 10,000 g for 10 min to remove cellular debris. For the inoculation assay and the enzyme-linked immunosorbent assay (ELISA), the clarified saliva was used after filter sterilization through a 0.22 µm Acrodisc filter (Pall Corporation, Ann Arbor, MI). After filtration, they were pooled and stored at −20°C until used.

### Preparation of immunoglobulin, amylase, and mucin

Lyophilized secretory Immunoglobulin A (sIgA) from human colostrum, α-amylase from human saliva, and mucin from bovine submaxillary glands (Sigma-Aldrich, St. Louis, MO) were mixed in PBS and adjusted to similar physiological concentrations as in human-saliva: 0.25, 0.4 and 2.7 mg/ml, respectively. These reagents were stored at −20°C until used.

### Bacterial sampling and colony-forming unit (CFU) estimate

Bacterial inoculation, sampling and CFU estimates were performed using procedures and conditions described previously [31], [34], [35]. All oral streptococci were cultured in BHI broth overnight and then washed twice with sterile phosphate-buffered saline (PBS). Our previous study demonstrated that colony counts of *S. mutans* were significantly higher than that of other streptococci (i.e. *S. sanguis, S. sobrinus*, and *S. salivarius*) in mice that ingested 1% sucrose in water one day before inoculation [31]. Thus, mice were given drinking water containing 1% sucrose (less than the usual concentration in juice) one day prior to *S. mutans* inoculation to reproduce the early adherence of *S. mutans* in conditions resembling a natural state. Chlorhexidine (0.2%) soaked sterile cotton swabs were used to disinfect the oral cavities of the mice including the maxillary incisor teeth. The cavity was immediately washed with sterile PBS. Four or 6 mice were treated with 100 µl of human saliva or salivary components for 2.5 min with the aid of micropipette. Casein was used as a control as a non-salivary component for the treatment. Five min after treatment, mice were washed with 100 µl of PBS. *S. mutans* solutions were introduced to the oral cavities of all females at 4 months of age at a final concentration of 7×10^9^ CFU in 250 µl of PBS during 2.5 min. Mice were separated into four groups based on the feeding conditions 24 h after inoculation. During the 24 h, one group was fed food with distilled water compared to another fed food with 1% sucrose-water; and the other set was food-deprived with 1% sucrose water or distilled water. Following inoculation, samples were collected from the labial surfaces of the maxillary incisor teeth with a sterile cotton ball and then dipped in 2 ml of PBS. To evaluate NOD/SCID.*e2f1* ^−/−^ mice as compared with previous results and to obtain stable data, samples collected from incisors were tested as parameters used in previous studies [31], [35]. The samples in sterile PBS were sonicated using ultrasonic dispersion (power output, 60 W) for 10 s, and then poured onto Mitis-Salivarius agar plates containing 0.02 M bacitracin (MSB). CFUs were determined by counting rough-surface colonies on MSB plates after 48 h using anaerobic incubation at 37°C.

### ELISA

To determine if sIgA reacts with *S. mutans* in vitro and if sIgA is absorbed on the tooth surface after treatment with human saliva, ELISA was performed with some modifications as described previously [33]. 96-well microtiter H-plates (Sumitomo Bakelite, Tokyo, Japan) were coated overnight at 4°C with a culture of *S. mutans* or *A. naeslundii* (1 µl/ml) in Na_2_CO_3_ coating buffer at pH 9.6 and incubated at 4°C overnight. In the sandwich assay to detect absorbed sIgA, we used 1/1,000 mouse anti-human immunoglobulin A antibody (Sigma-Aldrich, St. Louis, MO). The bacteria and antibody were diluted in Na_2_CO_3_ coating buffer at pH 9.6 and incubated at 4°C overnight. The plates were washed with PBS containing 0.1% (v/v) Tween 20 (PBST); and blocked with 1% (wt/vol) skim milk in PBST for 1 h at 37°C. Excess skim milk was removed by washing three times with PBST. To determine the presence of sIgA on the tooth surface, the tooth surface was swabbed using a sterile cotton ball after treatment with human saliva, and the swabbed ball was soaked in 2 ml coating buffer and shaken for 1 min. A 100 µl aliquot of 0.25 mg/ml sIgA, human saliva, or the soaked sample was added to the wells and the plates were incubated for 1 h at 37°C. The wells were washed three times with PBST; and further incubated for 1 h at 37°C with 100 µl 1/1,000 alkaline phosphatase conjugated goat anti-human immunoglobulin A antibodies (Zymed Laboratories, South San Francisco, CA). After three washings with PBST, the bound antibodies were detected after the addition of 50 µl of 3 mg/ml para-nitrophenyl phosphate as a substrate and incubated for 30 min at 37°C. Absorbance at 405 nm (A_405_) was measured using a microplate reader (Multiskan Bichromatic; Laboratory Japan, Tokyo, Japan). The mean value for each sample was used to calculate the ELISA value: Abs_405_ × 100/t (t: time of reaction). Triplicate measurements were performed and means calculated with standard error.

### Removal of *S. mutans*-specific sIgA

To determine if specific sIgA is employed for *S. mutans* colonization on the tooth surface, an absorption procedure was performed to remove specific antibody against *S. mutans*. Solutions of 1 mg/ml sIgA in PBS were absorbed with 0.5 mg (dry weight)/ml whole cells of lyophilized *S. mutans* UA159 at 37°C for 1 h and then overnight at 4°C. The mixture was centrifuged at 8,000 rpm for 10 min to remove *S. mutans*-IgA complex. Protein concentrations in the sIgA sample were measured using the Bio-Rad Protein Assay kit (Bio-Rad Laboratory, Hercules, CA) based on the method of Bradford and measured at 595 nm. The concentration of sIgA was adjusted to 0.25 mg/ml after the absorption procedure.

### Inhibiting effects of FruA in biofilm formation with *S. mutans*

To determine if the animal model could be used for the analysis of inhibitors for colonization and biofilm formation of *S. mutans* on the tooth surface, fructanase (FruA), a candidate inhibitor for biofilm formation of *S. mutans* [36], was used in the *in vivo* assay. The inhibiting activity of FruA at 1.25 units/ml was assayed in 96 well microtiter plates coated with human saliva [36]. FruA at 1.25 units/ml was also added within a 1% sucrose solution in drinking water (DW). FruA does not digest sucrose at 20∼25°C in 1% sucrose drinking water and does at 37°C in the oral cavity after mice drink the water [36]. After pre-treatment of sIgA following bacterial inoculation, all NOD/SCID.*e2f1* ^−/−^ mice were fed and supplied 1% sucrose water containing or not containing FruA. After 24 h inoculation, samples were collected and the CFU was counted as described above.

### Statistical analyses

The CFU and ELISA data were expressed as means ± standard deviations. GraphPad Prism version 5.0 d for Mac OS X (GraphPad Software, San Diego, CA) was used to perform tests of significance. The statistical significance of differences between two groups was determined using the unpaired *t*-test. For comparison between multiple groups, one-way analysis of variance (ANOVA) and Tukey-Kramer tests were used. P-values less than 0.001, 0.01 or 0.05 were considered statistically significant using two-tailed comparisons. All experiments were repeated and analyzed independently.

## Results

### Colonization of *S. mutans* in mice treated with human saliva

**Figure 1:**
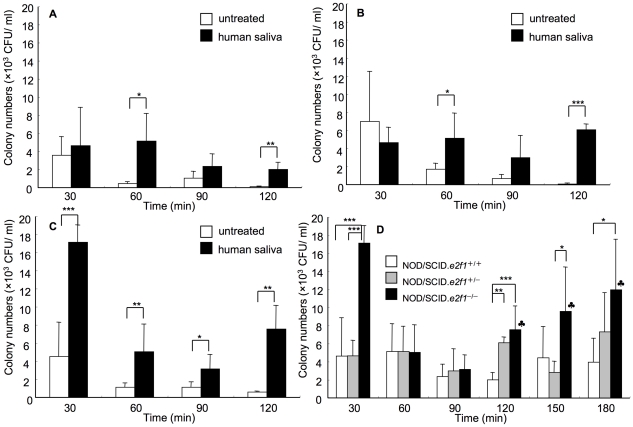
Colonization of *S. mutans* in human-saliva treated mice. Colony numbers of *S. mutans* in (A) NOD/SCID wild type, (B) NOD/SCID.*e2f1* ^+/−^, (C) NOD/SCID.*e2f1* ^−/−^ female mice, and 4 months of age pre-treated with and without human saliva prior to bacterial inoculation. Asterisks show significant differences (vs. untreated group, *P<0.05, ** *P*<0.01, *** *P*<0.001). (D) Time-course analysis of *S. mutans* colonization for each mouse strain pre-treated with human saliva prior to bacterial inoculation. Data were obtained from three independent experiments with 4 mice from each strain, and values are expressed as the means ± standard daviations (SDs) of the data (*P<0.05, ** *P*<0.01, *** *P*<0.001, represents significant differences vs. 90 min, *P*<0.05).

Human saliva is thought to play a significant role in the attachment of *S. mutans* to the tooth surface. We evaluated human saliva in bacterial colonization of NOD/SCID wild type, NOD/SCID.*e2f1* ^+/−^ mice, and NOD/SCID.*e2f1* ^−/−^ mice. *S. mutans* colonization in each mouse was significantly increased at all time points after the inoculation when they were treated with human saliva (Fig. 1 A, B and C). Bacterial numbers on the tooth surfaces were significantly higher in NOD/SCID.*e2f1* ^−/−^ mice than those in NOD/SCID wild type or NOD/SCID.*e2f1* ^+/−^ mice after 90 and 120–180 min post inoculation (Fig. 1 D). Colony numbers of *S. mutans* gradually decreased from 30 min to 90 min; however, after the colonization phase, the CFU gradually increased from 90 to 180 min in human saliva-treated NOD/SCID.*e2f1* ^−/−^ mice; whereas the other mice did not show a difference comparing time points.

### Effects of human saliva and salivary components in *S. mutans* colonization

**Figure 2:**
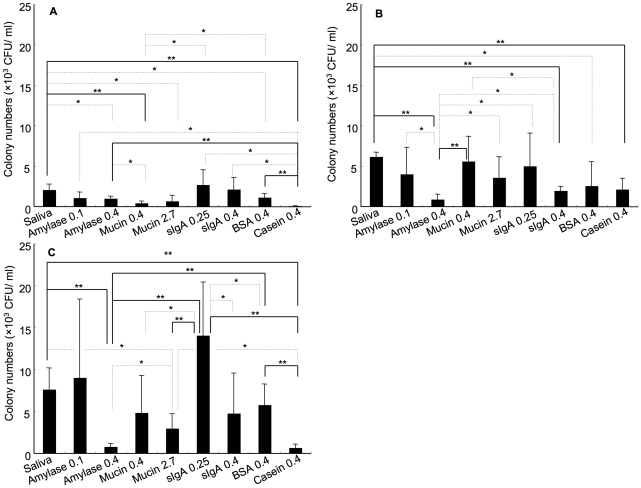
Effects of human saliva and salivary components in *S. mutans* colonization. Colony numbers of *S. mutans* in (A) NOD/SCID wild type, (B) NOD/SCID.*e2f1* ^+/−^, (C) NOD/SCID.*e2f1* ^−/−^ female mice, 4 months of age, at 120 min after inoculation. All mice were pre-treated with human saliva or salivary components prior to bacterial inoculation. Data are expressed as the means ± SDs of the results for 6 mice per strain (*P<0.05, ** *P*<0.01).

To determine if salivary components induce colonization of *S. mutans* on the tooth surface using the *in vivo* model, α-amylase, mucin and sIgA, receptors for *S. mutans* adhesins, were used to treat teeth before bacterial inoculation. CFUs were lower within non-treated mice compared to NOD/SCID.*e2f1* ^+/−^ and ^−/−^ 18 mice treated with all components other than casein treatment (control; non-salivary component) in NOD/SCID.*e2f1* ^+/+^ mice (data not shown). NOD/SCID.*e2f1* ^−/−^ mice had a higher colonization than NOD/SCID.*e2f1* ^+/−^ and NOD/SCID.*e2f1* ^+/+^ in each pre-treatment using the salivary components (Fig. 2 A, B and C). Bacterial colonization on teeth treated with 0.25 mg/ml sIgA at physiological concentrations was increased significantly in NOD/SCID.*e2f1* ^−/−^ mice (13,992±6,423); however, there was no significant difference in treating with saliva compared to sIgA (Fig. 2 C). In NOD/SCID.*e2f1* ^+/+^ and NOD/SCID.*e2f1* ^+/−^ mice, treatment with 0.25 mg/ml sIgA did not show greater colonization (Fig. 2 A, B). Further, higher concentrations of sIgA (0.4 mg/ml) did not result in higher colonization by *S. mutans* in comparison with BSA and casein in NOD/SCID.*e2f1* ^+/−^ and NOD/SCID.*e2f1* ^−/−^ mice (Fig. 2 B and C). Treatment in NOD/SCID.*e2f1* ^−/−^ mice with mucin (at 0.4 and 2.7 mg/ml) or with BSA did not result in increased levels of *S. mutans* colonization; these pre-treatments yielded significantly lower CFU counts compared to treatment with 0.25 mg/ml sIgA and considerably higher counts compared to treatment with 0.4 mg/ml amylase. Treatment with amylase at 0.1 mg/ml showed significantly higher colonization than at 0.4 mg/ml in NOD/SCID.*e2f1* ^+/−^; whereas there was no significant difference using NOD/SCID.*e2f1* ^−/−^ mice.

**Figure 3:**
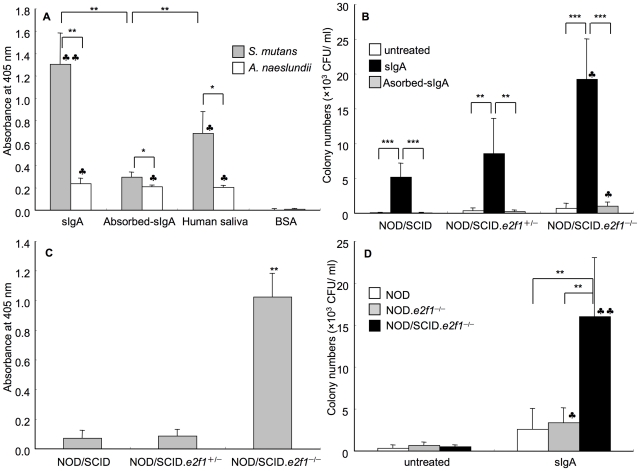
Effect of sIgA in *S. mutans* colonization. (A) Measurement of sIgA, absorbed sIgA and human saliva with *S. mutans*. BSA was the control. The ELISA results are expressed as the mean ± SD of absorbance obtained in three independent experiments. (Significant differences vs BSA, *P*<0.01, *P*<0.001). (B) Colonization assay on the tooth surface from NOD/SCID.*e2f1* ^−/−^ mice pre-treated with sIgA and absorbed sIgA at 120 min after inoculation. Untreated mice were controls. The results are expressed as the mean ± SD of absorbance obtained in six mice independent experiments. (Significant differences vs NOD/SCID, *P*<0.05). (C) Confirmation of residual sIgA on the tooth surface using ELISA, at 120 min after treatment. The ELISA results are expressed as the mean ± SD of absorbance obtained in three independent experiments. (Significant differences vs NOD/SCID and NOD/SCID.*e2f1* ^+/−^, *P*<0.05). (D) Evaluation of antibody deficiency and decreased saliva in the colonization assay, at 120 min after inoculation. The pre-treatment of sIgA was compared with the untreated group. Data are expressed as the means ± SDs of the results for 6 mice per strain (Significant differences vs untreated group, *P*<0.01, *P*<0.001).

SIgA was taken from human colostrum, and therefore may include various antibodies to pathogens. To confirm whether sIgA reacts with *S. mutans*, ELISA was performed using *S. mutans*-coated 96 well microtiter plates. *A. naeslunidii* was also used for coating as another oral bacterium. 0.25 mg/ml sIgA reacted strongly with *S. mutans* but not *A. naeslundii* (Fig. 3 A). The specificity of sIgA was observed by absorption of specific antibody to *S. mutans* in pre-incubation using *S. mutans* whole cells within sIgA. The absorbed sIgA was used for the ELISA assay and showed no significant reactivity to *S. mutans* (Fig. 3 A). Using human saliva, specific antibody to *S. mutans* was also observed using the ELISA assay (Fig. 3 A). The 0.25 mg/ml absorbed sIgA was used for the colonization assay in NOD/SCID wild type, NOD/SCID.*e2f1* ^+/−^ and NOD/SCID.*e2f1* ^−/−^ mice, and the effect of absorbed sIgA was compared with 0.25 mg/ml non-absorbed sIgA in all mice. The absorbed sIgA did not increase colonization of *S. mutans* in comparison with non-absorbed sIgA at 120 min after inoculation of *S. mutans* (Fig. 3 B). Therefore, increased colonization of *S. mutans* was dependent on specific antibody to *S. mutans* in sIgA and human saliva using this animal model. To determine whether sIgA remained on the tooth surface after treatment with human saliva, the surface was swabbed using a sterilized cotton ball at 120 min after the treatment in mice; and sIgA in the swabbed sample was measured using ELISA. The level of human-IgA that remained on the teeth for 120 min was significantly higher in NOD/SCID.*e2f1* ^−/−^ mice as compared to the other two strains (Fig. 3 C). This shows that specific sIgA antibody to *S. mutans* remains on the tooth surface after treatments with sIgA and human saliva in mice having decreased saliva and lack of IgA and IgG, the NOD/SCID.*e2f1* ^−/−^ mice. To determine whether a lack of IgA, by inserting the SCID type in NOD.*e2f1* ^−/−^ mice, promoted the colonization of *S. mutans*, the parent strain (NOD.*e2f1* ^−/−^ mice) and previous the parent strain (NOD mice) to NOD.*e2f1* ^−/−^ mice were used for the colonization assay after pre-treatment with 0.25 mg/ml sIgA and compared with NOD/SCID.*e2f1* ^−/−^ mice. We found that the colonization at 120 min after inoculation was significantly lower in NOD and NOD.*e2f1* ^−/−^ mice than NOD/SCID.*e2f1* ^−/−^ mice (Fig. 3 D). Therefore, lack of IgA and decreased saliva allowed specific IgA to remain on the tooth surface and to promote colonization of *S. mutans* in NOD/SCID.*e2f1* ^−/−^ mice.

### Synergistic effects of sucrose water and diet, and human saliva on *S. mutans* long-term colonization

**Figure 4:**
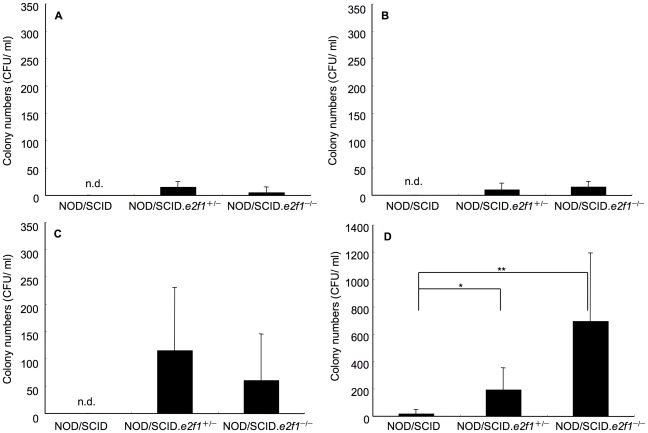
Comparison of different diet groups in *S. mutans* colonization. (A) group was not supplied with water or diet; (B) group was supplied with only 1% sucrose water; (C) group was supplied with both water and diet; (D) group was supplied with both 1% sucrose-containing water and diet. Samples were collected at 24 h after inoculation in NOD/SCID, NOD/SCID.*e2f1* ^+/−^ and NOD/SCID.*e2f1* ^−/−^ mice pre-treated with human saliva. Data are expressed as the means ± SDs of the results for 6 mice per strain (*P<0.05, ** *P*<0.01).

Long-term colonization is necessary in a mouse model to study several agents for the prevention to oral diseases. We observed that after inoculation, the colonization of *S. mutans* was slight at 24 hours in NOD/SCID, NOD/SCID.*e2f1* ^+/−^ and NOD/SCID.*e2f1* ^−/−^ mice pre-treated with human saliva (Fig. 4 A). Drinking water and diet including sucrose helped biofilm formation in other studies [14], [31]. A low concentration of 1% sucrose water was selected and supplied as drinking water with the usual animal diet for mice to establish an animal model that avoided high sucrose concentration-dependent colonization. The significant colonization was not observed in only the 1% sucrose water group as compared to that in non-sucrose water and non-diet group (Fig. 4 A, B). However, the group supplied with the combination of 1% sucrose-water and diet showed the most CFU/ml of *S. mutans*; colony numbers in NOD/SCID.*e2f1* ^−/−^ (693±500 CFU/ml) and in NOD/SCID.*e2f1* ^+/−^ (193±190) were significantly higher than those in wild type mice (17±32) (Fig. 4 D). The colonization was significantly higher in 1% sucrose-water and diet than non-sucrose water and diet in NOD/SCID.*e2f1* ^−/−^ mice (Fig. 4 C, D).

### Inhibition effects by FruA on colonization of *S. mutans* in vivo

In our previous report, purified and commercial fructanase (FruA) from *Aspergillus niger* completely inhibited *S. mutans* GS-5 biofilm formation on saliva-coated polystyrene and hydroxyapatite surfaces [36]. Therefore, we examined inhibition using FruA in *S. mutans* colonization in the established mouse model system. The bacterial load in NOD/SCID.*e2f1* ^−/−^ mice pre-treated with sIgA and supplied sucrose-water containing FruA (13±20 CFU/ml) decreased as compared to that without FruA (104±159); however, there was no significant difference (*P* = 0.088).

## Discussion

In this study we demonstrated homozygous E2F-1-deficient NOD/SCID (NOD/SCID.*e2f1* ^−/−^) mice are highly susceptible to *S. mutans* colonization when NOD/SCID.*e2f1* ^−/−^ mice are pre-treated with human saliva or sIgA using a low concentration (1%) sucrose supplement (Fig. 1 D, Fig. 4 D). The colonization levels were remarkably higher in NOD/SCID.*e2f1* ^−/−^ mice than other mouse strains including commercial strains: C57BL/6, B10.D2 and NOD mice [31], [37]. The high *S. mutans* susceptibility in NOD/SCID.*e2f1^−/−^* mice may be explained because of impaired salivary clearance. The systemic dysfunction of the salivary gland (e.g., enlarged nuclear size, increased numbers of ducts) caused by the E2F-1 deficiency is the principal reason for the decrease of saliva volume in the mice [33]. Previously we showed that the percent inhibition of saliva production volume (µl/100 g BW) in NOD/SCID.*e2f1^−/−^* mice was higher than that in other NOD-background mice [33], [35], [37].

Salivary component molecules that agglutinate bacteria include sIgA, mucins, parotid agglutinin, lysozyme, β_2_-microglobulin, and Ca^2+^ ions [38]. Some reports suggest that salivary components may promote colonization of certain strains of bacteria [8], [39]. Here we show the positive and negative effects of exogenous human salivary components in *S. mutans* colonization on the tooth surface. In particular, 0.25 mg/ml sIgA promoted colonization of *S. mutans* as compared to mucin, α-amylase, and others. SIgA is the predominant immunoglobulin found in all mucosal secretions including saliva. In general, sIgA is thought to participate in the local disposal of environmental antigens in the oral cavity [38]. Indeed, the inhibitory effects of sIgA against bacterial biofilm formation are well demonstrated [40], [41]. However, conversely in this study, sIgA played a role in aiding the colonization of *S. mutans* onto the tooth surface.

Physiological concentrations of amylase, mucin, and sIgA in human saliva are 0.4, 2.7 and 0.25 mg/ml respectively. Amylase at 0.4 mg/ml and 2.7 mg/ml mucin showed significantly lower colonization by *S. mutans* than 0.25 mg/ml sIgA, which showed higher colonization than human saliva treatment in NOD/SCID.*e2f1^−/−^* mice (Fig. 2). We considered that sIgA supported the attachment because specific sIgA against *S. mutans* was associated with the colonization. The activities of human saliva for colonization show dependency on specific sIgA (Fig. 3). In contrast, higher concentrations (0.4 mg/ml) of sIgA than physiological concentrations showed inhibiting activities as compared to physiological concentrations. The negative effects are also indicated by the effects of specific sIgA antibody on attachment. Therefore, multiple effects of specific sIgA may be dependent on sIgA concentration. The antibody titer to surface protein antigen from *S. mutans* was negatively correlated with the numbers of *S. mutans* in saliva from humans and mice [40], [42], [43]. The concentration of absorbed sIgA may be an important step for the colonization of *S. mutans* on the tooth surface and regulates the microbial flora in the oral cavity. Hapfelmeier *et al.* recently report reversible microbial colonization in germ-free mice during a dynamic IgA immune response [44]. They indicated the intestinal IgA system lacks classical immune memory characteristics; the intestinal IgA repertoire is characterized by constant attrition and thus represents the dominant species currently present in the intestine. In the oral cavity, a similar function of IgA production to intestinal IgA may cause and control commensal microbial flora. Our findings also show the dynamics of sIgA immune response, and sIgA may function to equalize the bacterial numbers in the oral cavity for the continuous presence of commensal oral bacteria.

Our *in vivo* colonization mouse system has a number of advantages to study specific sIgA effects because sIgA was absorbed on the tooth surface after exposure of sIgA to NOD/SCID.*e2f1^−/−^* mice. In our previous report, the production of protein per minute in 1 µl of saliva was significantly lower in NOD/SCID.*e2f1^−/−^* mice as compared to NOD/SCID mice [33]. NOD/SCID.*e2f1^−/−^* mice lack mature immunoglobulins due to severe combined immunodeficiency in NOD.*e2f1^−/−^* mice and a decreased volume of saliva as compared to both parent strains; NOD and NOD/SCID mice [33]. Therefore, sIgA was easily absorbed without competition with mouse IgA, and by decreasing the supply of proteins and poor salivary clearance on the tooth surface in NOD/SCID.*e2f1* ^−/−^ mice as compared to NOD.*e2f1* ^−/−^ mice [32] and NOD/SCID mice (Fig. 3 C). Further, absorbed specific sIgA against *S. mutans* was responsible for the colonization of *S. mutans* (Fig. 3 D). We show sIgA from human colostrum included sIgA against various microorganisms including *S. mutans*. Therefore, exposure of specific sIgA in the oral cavity may induce the first colonization and initial attachment of bacteria.

The effect of specific sIgA did not persist with the colonization over a long-term and as a result showed small numbers of *S. mutans* at 24 hours after inoculation, enough time to construct the biofilm on the tooth surface. SIgA supports attachment of *S. mutans*, but its effect was limited in the natural condition exposed with commensal bacteria and saliva in the oral cavity. Therefore, the sucrose water and diet were given as nutrients for *S. mutans* biofilm formation. Using 1% sucrose water and the usual mouse diet after inoculation supported long-term colonization in NOD/SCID.*e2f1* ^−/−^ and NOD/SCID.*e2f1* ^+/−^ mice in comparison to NOD/SCID mice (Fig. 4 D). We demonstrated that a concentration of 1% sucrose in drinking water with non-sucrose diet could induce significant colonization at 24 hours after inoculation. This shows the solid diet without sucrose enhanced colonization in combination with 1% sucrose drinking water (Fig. 4 B and D). The diet contains a few other carbohydrates, and carbohydrates in food debris or sucrose involved in debris absorbed with sucrose water after eating the diet and drinking for 24 hours may be employed in the production of the biofilm matrix. This was not observed previously using animal models for *S. mutans* infections. Possibly this biofilm formation closely resembles the natural environment of the oral cavity when humans consume various foods. In previous reports, conditions were dependent on excessive insoluble glucan formation in high sucrose water [45]–[48]. Their data showed rapid insoluble glucan formation and they likely generated these extreme effects under the high-sucrose experimental conditions favorable for production of biofilm. Humans eat a variety of foods, but they consciously control the oral condition to maintain oral health and view control of the intake of sucrose as very important. Therefore, we propose that the mouse model system observed here is more representative of the normal human oral environment; and better than previous model systems utilized for demineralization studies.

If this animal model system is used for assessment of various preventive dental caries agents, new preventative materials may be developed. Recently we reported fructanase (FruA) from *Streptococcus salivarius* and *Aspergillus niger* as a preventative. FruA can digest sucrose and prevent colonization [36], [49]. In this animal model, experiments using FruA in the mice supplied with 1% sucrose drinking water and diet at 24 hours after the inoculation, FruA inhibited the colonization by *S. mutans*; however, there were no significant differences (*p* = 0.088). It was considered that the animal model system may be useful in assessment of inhibiting agents recognized *in vitro*. However, the present system requires modifications to develop models for various oral infectious diseases as well as for dental caries. Our future studies will use this animal model to find inhibitory agents for infection by biofilm bacteria using the interaction of saliva, nutrients, and bacteria.
